# Detection of *Candida* DNA in peritoneal fluids by PCR assay optimizing the diagnosis and treatment for intra-abdominal candidiasis in high-risk ICU patients: A prospective cohort study

**DOI:** 10.3389/fmicb.2022.1070688

**Published:** 2023-01-05

**Authors:** Min Xie, Jin Shao, Zhe Wan, Ting Yan, Sainan Zhu, Shuangling Li, Jin Yu

**Affiliations:** ^1^Department of Critical Care Medicine, Peking University First Hospital, Peking University, Beijing, China; ^2^Department of Dermatology and Venerology, Peking University First Hospital, Beijing, China; ^3^Research Center for Medical Mycology, Peking University, Beijing, China; ^4^Beijing Key Laboratory of Molecular Diagnosis of Dermatoses, Peking University First Hospital, Beijing, China; ^5^National Clinical Research Center for Skin and Immune Diseases, Beijing, China; ^6^Department of Dermatology, Tianjin Academy of Traditional Chinese Medicine Affiliated Hospital, Tianjin, China; ^7^Department of Biostatistics, Peking University First Hospital, Peking University, Beijing, China

**Keywords:** intra-abdominal candidiasis, PCR assay, *Candida* spp., intensive care unit, peritoneal fluid

## Abstract

**Background:**

Intra-abdominal candidiasis (IAC) is the predominant type of invasive candidiasis with high mortality in critically ill patients. This study aimed to investigate whether the polymerase chain reaction (PCR) assay for detecting *Candida* DNA in peritoneal fluids (PF) is useful in diagnosing and management of IAC in high-risk patients in intensive care unit (ICU).

**Methods:**

A prospective single-center cohort study of surgical patients at high risk for IAC was conducted in the ICU. PF was collected from the abdominal drainage tubes (within 24 h) or by percutaneous puncture. Direct PF smear microscopy, PF culture, blood culture, and serum (1–3)-β-D-glucan were performed in all patients. For *Candida* PCR assay, the ITS1/ITS4 primers that targeted the ITS1-5.8 s-ITS2 regions were used for PCR, and sequencing analysis was used to identify the pathogen at the species level. IAC was defined according to the 2013 European consensus criteria.

**Results:**

Among 83 patients at high risk for IAC, the IAC criteria were present in 17 (20.5%). The sensitivity and specificity of the *Candida* PCR assay were 64.7 and 89.4%, respectively, and the area under the receiver operating characteristic curve was 0.77 (95% CI: 0.63–0.91). In this cohort, the positive predictive value and negative predictive value were 90.8% (95% CI: 80.3–96.2%) and 61.1% (95% CI: 36.1–81.7%), respectively. Diagnostic consistency was moderate (kappa 0.529, *p* < 0.001) according to the 2013 European consensus criteria.

**Conclusion:**

Detection of *Candida* DNA in PF using PCR can be considered an adjunct to existing routine diagnostic tools which may optimize the diagnosis and antifungal treatment of IAC in high-risk patients in the ICU.

## 1. Introduction

Intra-abdominal candidiasis (IAC) is the predominant type of invasive candidiasis (IC) accounting for 34–59% of IC (Leroy et al., 2009; Aguilar et al., 2015) with the high mortality rate is between 25 and 60% among critically ill patients (Delaloye and Calandra, 2014; Bassetti et al., 2015). The high mortality rate may be associated with the difficulty in early diagnosis of IAC (Pemán et al., 2017). Early diagnosis may lead to earlier antifungal therapy and therapeutic outcome improvement (Lagunes et al., 2017; Yan et al., 2020). However, appropriate tools for the early diagnosis of IAC are still lacking in critically ill patients. Traditional microbiological culture technology is time-consuming and has low sensitivity (Nguyen et al., 2012; Clancy and Nguyen, 2013; Fortún et al., 2014; Clancy and Nguyen, 2018). In comparison with microbiological cultures, non-culture-based technology (NCBT) could be considered a valuable tool for early diagnosis of IAC in recent years. However, most studies on NCBT have focused on bloodstream infections (candidemia). At the same time, very limited data are available on the clinical utility of NCBT targeting *Candida* DNA in peritoneal fluids (PF) in IAC. Existing experience mainly draws from *Candida* polymerase chain reaction (PCR) in blood samples in IC (Avni et al., 2011; Nguyen et al., 2012; Clancy and Nguyen, 2018). However, in a population with a low proportion of candidemia, especially for IAC without candidemia, the performance of *Candida* PCR using blood samples is still controversial (León et al., 2016; Fortún et al., 2020). Corrales et al. (2015) explored the utility of *Candida* PCR in PF of patients with peritonitis. This study compared the accuracy of a PCR DNA low-density microarray system (CLART STIs B) with the BACTEC FX automated culture method for detecting *Candida* spp. in 161 PF samples. The overall agreement between the PCR assay and culture method was good, which demonstrated the potential clinical utility of *Candida* PCR in PF for diagnosing IAC. However, this study did not compare the agreement between *Candida* PCR in PF and current diagnostic criteria for IAC.

The European Society of Intensive Care Medicine and European Society of Clinical Microbiology and Infectious Diseases (ESICM/ESCMID) task force defined treatments using different terminology such as preventive therapies, preemptive therapies, empirical therapies, and target therapies according to the timing and the basis of initiation of antifungal treatment in the practical management of IC in critically ill patients (Martin-Loeches et al., 2019). The targeted therapies are treatments based on microbiological confirmation of invasive infection due to *Candida* species (e.g., a positive blood culture for *Candida* species), limited by the low sensitivity and long-term cost of traditional microbiological culture technology. Assuming that *Candida* PCR positivity could be included in the definition of microbiological confirmation, it is expected to optimize the antifungal treatment of IAC.

To complete these analyses, we conducted a prospective cohort study in critically ill surgical patients with a high risk of IAC to evaluate the clinical utility of *Candida* PCR in PF for IAC diagnosis and treatment.

## 2. Materials and methods

This was a single-center prospective cohort study. The study protocol was approved by the Clinical Research Ethics Committee of Peking University First Hospital (2018-153) and registered with China Clinical Trial Registration Center identifier (CHiCTR190022695). Written informed consent was obtained from the next of kin or legal representative of each participant.

### 2.1. Patients recruitment

All consecutive critically ill patients were screened at ICU admission. The inclusion criteria were adult patients (age 18 years or older); a diagnosis of secondary peritonitis; tertiary peritonitis or abdominal abscess; high risk of IAC; and an available abdominal drainage tube placed by surgery or percutaneous puncture. High risk of IAC was defined as either of the following conditions (Bassetti et al., 2013; Hall et al., 2013; Rhodes et al., 2017): (1) Recurrent gastrointestinal perforations, perforations untreated for more than 24 h, or both, (2) Recurrent abdominal surgery within 30 days, (3) Postoperative suspected or confirmed gastrointestinal anastomosis leakage, (4) Multifocal colonization of *Candida* spp., (5) Severe acute pancreatitis, and (6) Septic shock. Patients who met any of the following criteria were excluded: refused to participate; neutropenia (defined as total leukocyte count <1,000/mm^3^); human immunodeficiency virus (HIV) infection; receiving immunosuppressants or systemic steroids (prednisone equivalent ≥20 mg/day) within 7 days before ICU admission; Abdominal drainage tube placed more than 24 h prior; The patient had been enrolled within the ICU admission; and incomplete data collection.

### 2.2. Baseline and perioperative data

After obtaining written consent with trained researchers, data for each patient were collected through the electronic medical record system of the Peking University First Hospital. The baseline data included sociodemographic parameters, preoperative comorbidities, surgical diagnoses, and surgical type. The severity of comorbid diseases and general status was evaluated using age-adjusted Charlson comorbidity index (ACCI) and the American Society of Anesthesiology (ASA) physical status classification. Postoperative data included the Acute Physiology and Chronic Health Evaluation (APACHE) II score and sepsis-related organ failure assessment (SOFA) score, calculated for each patient within 24 h of ICU admission. Data on infection status included risk factors for IAC, main laboratory test results at ICU admission, and initial antifungal treatment. The infection characteristics in patients with IAC included anatomical location (source of IAC), initial and duration of antifungal therapy. The outcomes included length of stay (LOS) in the ICU and hospital, duration of mechanical ventilation, all-cause ICU mortality, 28-day mortality, and hospital mortality.

### 2.3. Microbiological cultures

The PF sample (10 ml) was extracted using a drainage tube placed for less than 24 h or by direct percutaneous puncture on the day of enrollment. The PF samples were divided into two equal parts and immediately sent to the laboratory, one of which was used for DNA extraction and molecular identification by PCR, and the other was kept at 4°C for microbiological studies.

Direct smear microscopy (PRE-V1 automatic Gram dyeing machine, Bio-Meriere, France) and microbiological culture (LRH-250A biochemical incubator, Taihong Medical Device Co., Ltd., China, culture period: seven days) in PF were performed within 24 h. Venous blood samples (20 ml) were extracted from at least two different puncture sites and added into both aerobic and anaerobic blood culture bottles (two bottles for each type, BD BACTECTM, United States) and immediately sent to the laboratory for blood culture (BACTEC FX Automatic blood culture instrument, BD BACTECTM, United States, culture period: seven days).

### 2.4. Quantitation of serum (1–3)-β-D-glucan

After participants enrollment, venous blood (4 ml) was extracted on the latest available date (Monday and Thursday) and immediately sent to the laboratory for centrifugation (3,000 rpm, 15 min) to separate the serum, which was stored at 4°C. BDG (GKT5M Dynamic Fungal Detection Kit, Beijing Jin Shan Chuan Science and Technology Development Co., Ltd., China) was assayed within 2 h and a serum BDG result >100 pg/ml was considered positive.

### 2.5. PCR assay

The DNA of the PF samples was extracted using the operation method of QIAamp DNA Mini Kit (QIAGE Company, Germany) according to the manufacturer’s instructions. The general primers ITS1 (5′-TCCGTAGGTGAACCTGCGG-3′) and ITS4 (5′-TCCTCCGCTTATTGATATGC-3′) were used for the PCR amplification (Paul and Kannan, 2019). The PCR products were sent to Sangon Biotech Co., Ltd. (Shanghai, China) for Sanger sequencing. The sequencing primers were the same as those used for amplification. All sequences were blasted against the National Center for Biotechnology Information (NCBI) database (https://blast.ncbi.nlm.nih.gov/Blast.cgi. Accessed January 31, 2021), and a similarity cutoff value of ≥99% was identified at the species level.

### 2.6. Definition criteria of IAC

The clinical physician diagnosed the patient with secondary peritonitis, tertiary peritonitis, or an abdominal abscess. IAC was defined according to the 2013 European Consensus criteria (Bassetti et al., 2013): (1) yeast detection by direct microscopy examination or growth in culture from purulent or necrotic intra-abdominal specimens obtained during surgery or by percutaneous aspiration; (2) *Candida* species (spp.) are cultured from bile, intra-biliary duct devices, and biopsy of intra-abdominal organs; (3) *Candida* spp. growth from blood cultures in the clinical setting of secondary and tertiary peritonitis in the absence of any other pathogen; and (4) *Candida* spp. growth from drainage tubes if placed less than 24 h before the cultures.

### 2.7. Antifungal treatment

The ESICM/ESCMID task force (Martin-Loeches et al., 2019) defined treatments using different terminologies such as preventive therapies, empirical therapies, preemptive therapies, and target therapies according to the timing and basis of initiation of antifungal treatment. Preventive therapies are antifungal therapies for critically ill patients with risk factors (such as immunosuppression), risk factors linked to the reason for ICU admission, or both. Empirical therapies refer to the administration of antifungal agents in patients with infection signs and symptoms along with specific risk factors for IC, irrespective of biomarkers. Preemptive therapies are antifungal treatments administered to patients at risk for IC, with a diagnosis based on fungal biomarkers. In this trial, the fungal biomarker tested positive for serum BDG levels. Targeted therapies are treatments based on microbiological confirmation of an invasive infection caused by *Candida* spp. In this study, microbiological confirmation refers to at least one positive result in the blood culture for *Candida* spp., PF direct microscopy examination for yeast or yeast-like fungal elements, and PF culture for *Candida* spp. Two independent researchers judged the diagnosis of IAC and classification of the initial antifungal treatment. Differences between the two researchers should be thoroughly discussed with the clinical physician to reach a final agreement.

### 2.8. Statistical analysis

Continuous variables were evaluated for normality using the Shapiro–Wilk test and are presented as the mean ± SD or median (interquartile range). Differences between means were compared using the Student’s t-test (normal distribution) or Mann–Whitney U test (non-normal distribution). The mean or median difference between the groups and the 95% CI of the difference were calculated. Categorical variables are presented as n (%) and were analyzed using the chi-squared test or Fisher’s exact test. The chi-squared test was used to compare sensitivity, specificity, negative predictive value (NPV), and positive predictive value (PPV) between *Candida* PCR assay and the 2013 European Consensus criteria as the gold standard. The area under the receiver operating characteristic curve (AUC ROC) analysis was used to evaluate the utility of *Candida* PCR for diagnosing IAC, and the kappa coefficient was used to evaluate the consistency between *Candida* PCR and the gold standard. After excluding the variables with collinearity, variables with a *p* < 0.15 in univariate analyses were included in a multivariate logistic model (backward) to identify IAC risk factors. All tests were two-sided. *p* < 0.05 were considered statistically significant. Statistical analysis was performed with the SPSSw Statistics 25.0 software package (IBMw Inc., Chicago, United States).

## 3. Results

**Figure 1:**
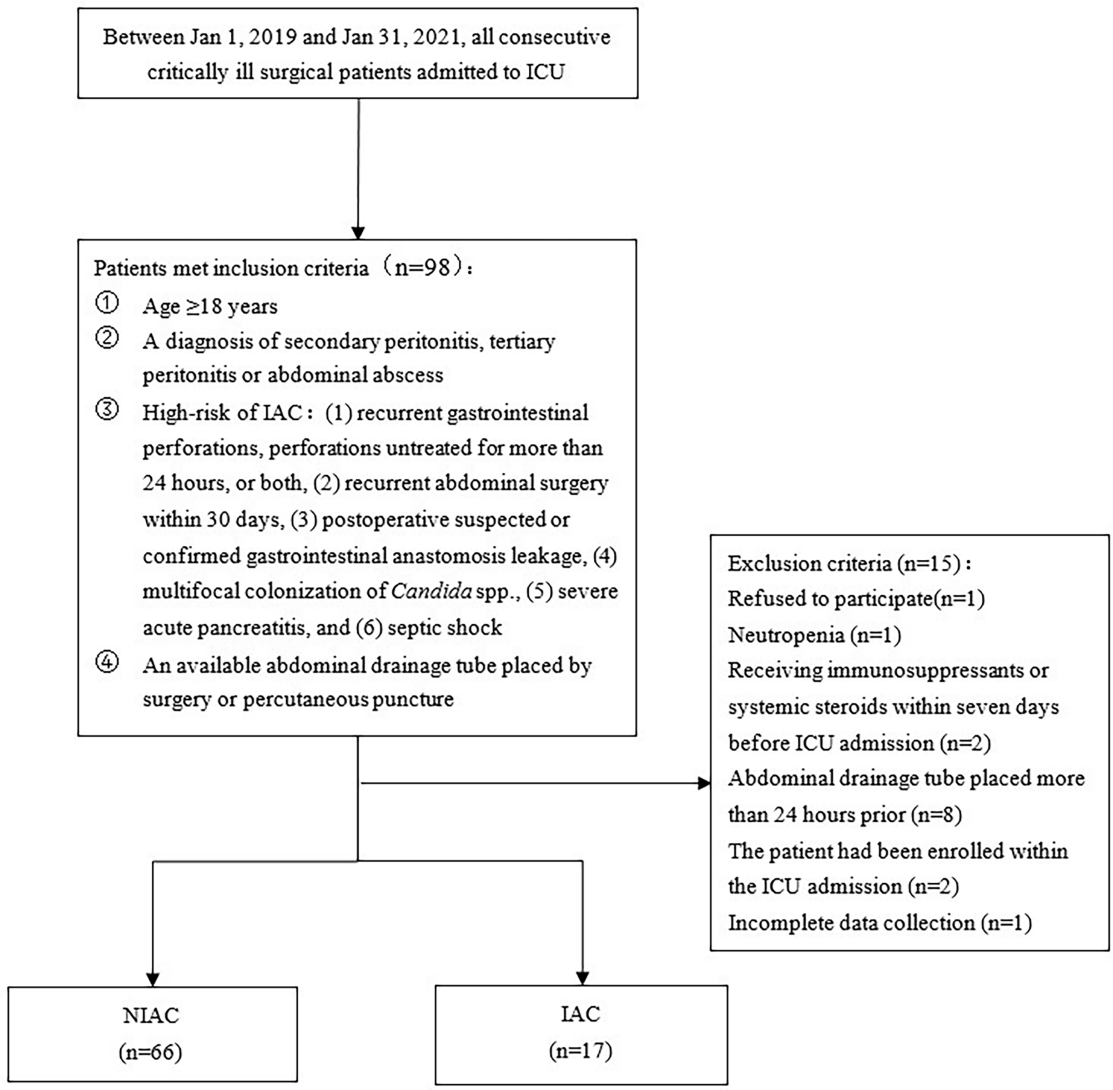
Flow chart. IAC, intra abdominal candidiasis.

A total of 98 patients were assessed for inclusion in the study from January 1, 2019, to January 31, 2021. The flowchart for recruiting patients was shown in Figure 1. Eighty-three patients at high risk of IAC were finally enrolled (Figure 1). A total of 17 patients were determined to have IAC. The incidence of IAC was 20.5% (17/83). There was no difference between the IAC and NIAC groups regarding baseline characteristics and high-risk factors for IAC (Table 1). Of the patients in the IAC group, 94.1% (16/17) received antifungal treatment, which was significantly higher than that of the NIAC group (56.1%, 37/56, *p* = 0.004, Table 2). Multivariate logistic regression showed that the surgical site involved in the upper gastrointestinal tract (OR 6.119, 95% *CI:* 1.635–22.896, *p* = 0.007) and the percentage of neutrophils >90% on ICU admission (OR 6.665, 95% *CI:* 1.612–27.412, *p* = 0.009) were independent risk factors for IAC in this cohort of patients (Table 3). No new high-risk factors for IAC were identified in upper and non-upper gastrointestinal tract subgroup (for details, see Supplementary Table 1).

**Table 1:** Baseline characteristics and high-risk factors for IAC.

	Total (*N* = 83)	NIAC (*N* = 66)	IAC (*N* = 17)	Value of *p*
Age (years)	71.2 ± 13.8	70.5 ± 14.5	74.1 ± 10.6	0.34
Male sex	50 (60.2)	36 (54.5)	14 (82.7)	0.051
Body mass index (kg/m^2^)	27.9 ± 5.4	28.5 ± 5.2	25.7 ± 5.6	0.056
**Preoperative comorbidity**				
Solid tumor	50 (60.2)	41 (62.1)	9 (52.9)	0.49
Diabetes mellitus	21 (25.3)	17 (25.8)	4 (23.5)	1
COPD	5 (6.0)	4 (6.1)	1 (5.9)	1
Hypertension	40 (48.2)	30 (45.5)	10 (58.8)	0.325
Coronary heart disease	13 (15.7)	10 (15.2)	3 (17.6)	0.724
Congestive heart failure	3 (3.6)	2 (3.0)	1 (5.9)	0.502
Arrhythmia	12 (14.5)	7 (10.6)	5 (29.4)	0.063
Abnormal renal function^a^	2 (2.4)	2 (3.0)	0 (0.0)	NA
Cerebrovascular disease	7 (8.4)	5 (7.6)	2 (11.8)	0.628
**High-risk factors for IAC, *n* (%)**				
Recurrent gastrointestinal perforations, perforations untreated for more than 24 h, or both	39 (47.0)	28 (42.4)	11 (64.7)	0.101
Recurrent abdominal surgery within 30 days	20 (24.1)	17 (25.8)	3 (17.6)	0.751
Postoperative suspected or confirmed gastrointestinal anastomosis leakage	21 (25.3)	16 (24.2)	5 (29.4)	0.756
Multifocal colonization by *Candida* spp.	1 (1.2)	1 (1.5)	0 (0.0)	/
Severe acute pancreatitis	2 (2.4)	2 (3.0)	0 (0.0)	/
Septic shock	46 (55.4)	38 (57.6)	8 (47.1)	0.437
ACCI	5 (4–6)	5 (4–6)	5 (3–6.5)	0.802

Results are presented as mean ± SD or median (interquartile range), or *n* (%). ^a^Serum creatinine >177 μmol/l. COPD, chronic obstructive pulmonary diseases; ACCI, age-adjusted Charlson comorbidity index.

**Table 2:** Perioperative variables and infection status.

	Total (*N* = 83)	NIAC (*N* = 66)	IAC (*N* = 17)	Value of *p*
**Surgical diagnosis**				0.462
Tumor	37(44.6)	30(45.5)	7(41.2)	
Gastrointestinal perforation without tumor	19(22.9)	13(19.4)	6(35.3)	
Postoperative gastrointestinal anastomosis leakage	12(14.5)	9(13.6)	3(17.6)	
Intestinal obstruction without tumor	5(6.0)	5(7.6)	0(0.0)	
Others^a^	10(12.0)	9(13.6)	1(5.9)	
**Site of abdominal surgery**				0.001
Upper gastrointestinal tract	23 (27.7)	13 (19.7)	10 (58.8)	
Non-upper gastrointestinal tract	60 (72.3)	53 (80.3)	7 (41.2)	
**Surgery type**				1.000
Emergency	64 (77.1)	51 (77.3)	13 (76.5)	
Elective	19 (22.9)	15 (22.7)	4 (22.9)	
ASA classification IV or V	34 (41.0)	29 (43.9)	5 (29.4)	0.277
**ICU admission**				
APACHE II score	15 (11–19)	15 (11–19)	17 (12–20)	0.432
SOFA score	6 (4–9)	6.5 (4–9)	6 (4–8.5)	0.75
Temperature, °C	37.4 (36.7–37.9)	37.4 (36.7–37.9)	37.5 (36.8–38.0)	0.631
WBC (×10^9^/L)	9.8 (6.8–14.1)	10.1 (8.0–14.1)	8.4 (4.4–14.4)	0.147
NE, %	89.5 (85.2–93.1)	88.2 (84.6–92.6)	92.0 (89.0–94.3)	0.037
PCT (ng/ml)^b^	3.8 (1.0–15.1)	4.0 (0.6–15.9)	3.2 (1.4–7.4)	0.909
CRP (ng/ml)^c^	133.3 (80.2–193.7)	142.0 (84.8–196.0)	69.3 (57.1–159.5)	0.088
Antifungal treatment	53 (63.9)	37 (56.1)	16 (94.1)	0.004
**Initial antifungal treatment**^**d**^				0.001
Preventive therapies	2/53 (3.8)	2/37 (5.4)	0/16 (0.0)	
Empirical therapies	42/53 (79.2)	33/37 (89.2)	9/16 (56.3)	
Preemptive therapies	3/53 (5.7)	2/37 (5.4)	1/16 (33.3)	
Target therapies	6/53 (11.3)	0/37 (0.0)	6/16 (37.5)	

Results are presented as mean ± SD or median (interquartile range), or *n* (%). ^a^Others include intestinal ischemia, acute pancreatitis, choledocholithiasis, acute appendicitis, intra-abdominal hemorrhage, and postoperative intra-abdominal infection; ^b^*N* = 80; ^c^*N* = 62; ^d^Two patients began antifungal treatment before percutaneous drainage, and one patient began antifungal treatment before ICU admission. ASA, American Society of Anesthesiology; APACHE, acute physiology and chronic health evaluation; SOFA, sepsis-related organ failure assessment; WBC, white blood cell, NE neutrophils; CRP, C-reaction protein; PCT, procalcitonin.

**Table 3:** Risk factors of IAC.

	Univariate logistics		Multivariate logistics	
	OR (95% *CI*)	Value of *p*	OR (95% *CI*)	Value of *p*
Age ≥75 years	1.004 (0.345–2.920)	/	/	0.995
Male	3.889 (1.012–14.819)	0.071	4.504 (0.877–23.117)	0.047
BMI >25 kg/m^2^	0.331 (0.109–1.007)	0.667	1.410 (0.295–6.735)	0.051
Upper gastrointestinal surgery	5.824 (1.862–18.221)	0.007	6.119 (1.635–22.896)	0.002
Emergency	0.956 (0.271–3.369)	/	/	0.944
ASA IV or V	0.532 (0.168–1.681)	/	/	0.282
Underlying diseases				
Solid tumor	0.686 (0234–2.009)	/	/	0.492
Diabetes mellitus	0.887 (0.254–3.093)	/	/	0.851
COPD	0.969 (0.101–9.276)	/	/	0.978
Hypertension	1.714 (0.582–5.051)	/	/	0.328
Coronary heart disease	1.200 (0.291–4.949)	/	/	0.801
Congestive heart failure	2.000 (0.170–23.461)	/	/	0.581
Arrhythmia	3.512 (0.953–12.947)	0.063	4.739 (0.920–24.416)	0.059
Cerebrovascular disease	1.627 (0.287–9.216)	/	/	0.582
APACHE II^a^	1.034 (0.943–1.135)	/	/	0.474
SOFA^a^	0.968 (0.812–1.155)	/	/	0.721
Temperature^a^, °C	1.083 (0.579–2.027)	/	/	0.803
WBC^a^, ×10^9^/L	0.939 (0.848–1.041)	/	/	0.231
NE% > 90% ^a^	3.692 (1.164–11.710)	0.009	6.665 (1.612–27.412)	0.027
PCT^a,b^, ng/ml	0.979 (0.925–1.036)	/	/	0.462
Recurrent gastrointestinal perforations, perforations untreated for more than 24 h, or both	2.488 (0.822–7.535)	0.26	2.144 (0.568–8.085)	0.107
Recurrent abdominal surgery within 30 days	0.618 (0.158–2.415)	/	/	0.489
Postoperative suspected or confirmed gastrointestinal anastomosis leakage	1.302 (0.398–4.261)	/	/	0.663
Septic shock	0.655 (0.225–1.910)	/	/	0.438

^a^At ICU admission; ^b^*N* = 80; BMI, body mass index; ASA, American Society of Anesthesiology; COPD, chronic obstructive pulmonary diseases; SOFA, sepsis-related organ failure assessment; APACHE, acute physiology and chronic health evaluation; WBC, white blood cell; NE, neutrophils; CRP, C-reaction protein; PCT, procalcitonin.

**Figure 2:**
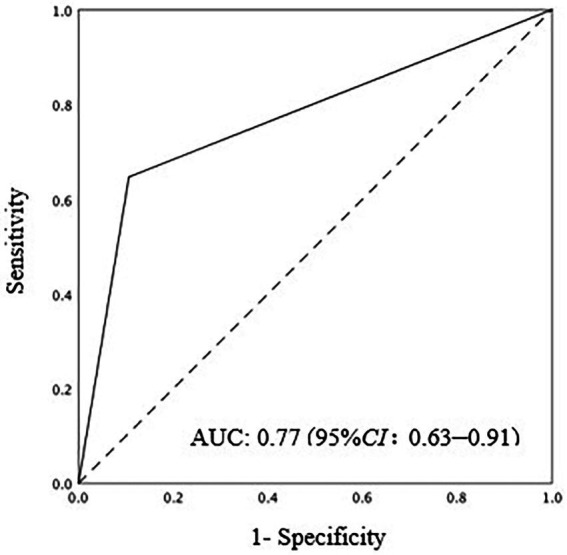
AUC ROC of positive *Candida* PCR in PF for IAC diagnosis. AUC ROC: 0.77(95%CI: 0.063–0.91). AUC ROC, area under the receiver operating characteristic curve; PCR polymerase chain reaction; IAC, intra abdominal candidiasis.

The sensitivity of *Candida* PCR in PF was 64.7% (95% *CI:* 38.6–84.7%), the specificity was 89.4% (95% *CI:* 78.8–95.3%), and the AUC of ROC was 0.77 (95% *CI:* 0.63–0.91; Figure 2). In high-risk patients with IAC, the NPV of *Candida* PCR in PF was 90.8% (95% *CI:* 80.3–96.2%) and the PPV was 61.1% (95% *CI:* 36.1–81.7%). The positive *Candida* PCR results in PF were moderately consistent with the 2013 European Consensus IAC diagnostic criteria (kappa 0.529, *p* < 0.001). Serum BDG has high specificity and a good NPV, but its sensitivity is low. The PPV of BDG only increased to 66.7% (95% *CI:* 12.5–98.2%) after two consecutive positive BDG results (Table 4). The consistency between the serum BDG test and the IAC diagnostic criteria was poor (kappa 0.098). Combining *Candida* PCR with the serum BDG test improved the PPV (from 40.0% [7.3–83.0%] to 50.0% [26.7–97.3%]); however, it did not help optimize the sensitivity and NPV (from 80.8% [70.0–88.5%] to 80.2% [69.6–88.0%]) or improve the consistency (kappa from 0.098 to 0.065) with the gold standard.

**Table 4:** Performances of *Candida* PCR and BDG used alone and combined for IAC diagnosis.

	Sensitivity% (95%*CI*)	Specificity% (95%*CI*)	NPV% (95%*CI*)	PPV% (95%*CI*)	Kappa	Value of *p*
Positive *Candida* PCR	64.7 (38.6–84.7)	89.4 (78.8–95.3)	90.8 (80.3–96.2)	61.1 (36.1–81.7)	0.529	<0.001
First positive BDG^a^	11.8 (2.1–37.7)	95.5 (86.4–98.8)	80.8 (70.0–88.5)	40.0 (7.3–83.0)	0.098	0.264
Two consecutive positive BDG^a,b^	18.2 (3.2–52.2)	96.2 (78.4–99.8)	73.5 (55.3–86.5)	66.7 (12.5–98.2)	0.181	0.144
Positive *Candida* PCR combining with first positive BDG^a^	5.9 (0.3–30.8)	98.5 (90.7–99.9)	80.2 (69.6–88.0)	50.0 (26.7–97.3)	0.065	0.295
Positive *Candida* PCR combining with two consecutive positive BDG^a,b^	9.1 (0.5–42.9)	100.0 (84.0–100.0)	72.2 (54.6–85.2)	100.0 (5.5–100.0)	0.123	0.119

Results are presented as *n* (%). PCR, polymerase chain reaction; BDG, (1–3)-ß-D-glucan; IAC, intra-abdominal candidiasis. ^a^BDG >100 pg/ml was considered positive; ^b^37 patients underwent two consecutive BDG tests.

*Candida albicans* was the most frequent isolated species in the PF culture [8(47.1%)]. Other *Candida* spp. mainly included *C. glabrata* (3 [17.6%]), *C. tropicalis* (3 [17.6%]), and *C. krusei* (1 [5.9%]). In 17 PF samples from the IAC group, *Candida* PCR was positive in 11 cases (64.7%), including 4 of *C. albicans* (23.5%), 4 of *C. glabrata* (23.5%), 2 of *C. tropicalis* (11.8%), and 1 of *C. krusei* (5.9%); 6 cases were negative. A total of 7 cases were positive for PCR in PF, but negative for PF direct microscopy examination, PF culture, and blood culture. Of these, 5 patients received antifungal therapy with echinocandins (Caspofungin in 4 cases and Micafungin in 1 case). In one patient, the clinician initiated antifungal treatment on the basis of positive BDG, which was preemptive therapy. The remaining 4 cases were empirical therapies. The results of DNA identification by PCR and culture were consistent in 10 of 11 samples with positive PCR results (Table 5). In the inconsistent sample, DNA identification by PCR was identified as *C. glabrata* but cultured as *C. glabrata* and *C. tropicalis*. In addition, two cases with negative PF cultures showed positive PCR results (one case each of *C. albicans* and *C. glabrata*).

**Table 5:** Characteristics of infection in patients with IAC (*N* = 17).

	N (%) or median (interquartile range)
**Candidemia**	2 (11.8)
*Candida albicans*	1 (5.9)
*Candida glabrata*	1 (5.9)
**Bacteremia**	1 (5.9)
**Bacterial culture in PF**	
Gram-negative bacilli	6 (35.3)
Gram-positive cocci	6 (35.3)
No bacterial isolation	7 (41.2)
**Broad-spectrum antibiotic therapy**	17 (100.0)
**Fungal culture in PF**^**a**^	
*Candida albicans*	8 (47.1)
*Candida glabrata*	3 (17.6)
*Candida tropicalis*	3 (17.6)
*Candida krusei*	1 (5.9)
No fungal isolation	3 (17.6)
**Fungal smear microscopy in PF**	
Positive	2 (11.8)
Negative	15 (88.2)
***Candida* spp. identification by PCR in PF**	
*Candida albicans*	4 (23.5)
*Candida glabrata*	4 (23.5)
*Candida tropicalis*	2 (11.8)
*Candida krusei*	1 (5.9)
No fungal isolation	6 (35.3)
**Initial antifungal drugs**^**b**^	
Micafungin	7 (41.2)
Caspofungin	7 (41.2)
Voriconazole	1 (5.9)
Fluconazole	1 (5.9)
**Duration of antifungal therapy (days)**	4 (0–10)

Results are presented as *n* (%) or median (interquartile range). IAC, intra-abdominal candidiasis; PF, peritoneal fluids. ^a^Both *Candida glabrata* and *Candida tropicalis* were reported in fungal culture in one IAC patient; ^b^One patient with IAC died before antifungal therapy was initiated.

The clinical characteristics of the 18 patients with positive PCR results for PF are presented in Table 6. The positive rate of PCR for PF was 21.7% (18/83), while the positive rate of current IAC diagnostic methods was 20.5% (17/83). If positive PCR results for PF were included in the confirmed diagnostic criteria, the number of confirmed diagnoses of IAC in 83 patients would increase to 24, and the incidence of IAC would increase from 20.5 to 28.9% (*p* < 0.001). Assuming that the positive PCR in PF was one of the microbiologically confirmed types of evidence of targeted therapies, the classification of initial antifungal treatment would change in some patients receiving antifungal treatment. The number of patients receiving targeted therapies would increase from 6 to 17, and the proportion of targeted therapies in the initial antifungal treatment would increase from 11.3 to 32.1% (*p* < 0.001; Table 7).

**Table 6:** The clinical characteristics of the patients with positive PCR results for PF (n = 18).

No.^a^	Age	Sex	Group	Candidemia	Direct smear microscopy in PF	PF culture	*Candida* DNA identification by PCR in PF^b^	Serum BDG (pg/ml)	Initial antifungal drugs	Survival in ICU
						Bacteria	Fungi			
1	78	F	NIAC	No	NF	NF	NF	*Candida albicans*	189.7	Caspofungin
2	89	M	IAC	No	NF	NF	*Candida albicans*	*Candida albicans*	<10	Caspofungin
3	71	M	IAC	NP	NF	*Enterococcus faecalis*	*Candida albicans*	*Candida albicans*	<10	Caspofungin
4	78	F	NIAC	No	NF	*Acinetobacter baumanii*	NF	*Candida parapsilosis*	<10	Caspofungin
5	71	M	NIAC	No	GPC	*Pseudomonas aeruginosa, Enterococcus faecalis*	NF	*Candida lusitaniae*	<10	Micafungin
6	83	M	IAC	No	NF	*Klebsiella pneumoniae*	*Candida glabrata*	*Candida glabrata*	59.9	Micafungin
7	64	M	NIAC	No	NF	*Escherichia Coli, Pseudomonas aeruginosa, Enterococcus faecalis*	NF	*Candida parapsilosis*	<10	Caspofungin
8	72	M	IAC	No	Fungal spores, GNB, GPC	NF	*Candida krusei*	*Candida krusei*	<10	Caspofungin
9	48	M	NIAC	No	NF	*Escherichia Coli*	NF	*Candida albicans*	28.8	Caspofungin
10	87	M	NIAC	NP	NF	NF	NF	*Candida tropicalis*	43.3	No
11	79	M	IAC	No	NF	NF	*Candida glabrata, Candida tropicalis*	*Candida glabrata*	<10	No
12	82	M	IAC	No	GPC	*Staphylococcus aureus*	*Candida albicans*	*Candida albicans*	135.2	Micafungin
13	74	F	IAC	No	NF	NF	*Candida tropicalis*	*Candida tropicalis*	<10	Caspofungin
14	89	F	NIAC	No	NF	*Klebsiella pneumoniae*	NF	*Candida albicans*	<10	No
15	66	M	IAC	No	NF	NF	*Candida glabrata*	*Candida glabrata*	<10	Micafungin
16	63	M	IAC	Yes	GPC	*Enterococcus faecalis, Staphylococcus aureus*	NF	*Candida glabrata*	<10	Caspofungin
17	78	M	IAC	No	GBN, GPC	*Enterococcus avium, Citrobacter freundii*	*Candida tropicalis*	*Candida tropicalis*	26.6	Caspofungin, AmB
18	74	F	IAC	NP	Fungal spores, GNB	*Enterobacter cloacae*	NF	*Candida albicans*	<10	Caspofungin

^a^In this table, No. 1.4.5.7.9.10 and 14 patients with positive *Candida* PCR were in NIAC group; No. 16 patient was in IAC group because of candidemia; No. 18 patient was in IAC group because of fungal spores in direct smear microscopy in PF. ^b^GenBank accession numbers for the nucleotide sequences were OP418489-OP418506. PCR, polymerase chain reaction; PF, peritoneal fluids; IAC, intra-abdominal candidiasis; BDG, (1–3)-ß-D-glucan; GPC, Gram-positive cocci; GNB, Gram-negative bacilli; NF, not found; NP, not performed; AmB, amphotericin B.

**Table 7:** Incidence and initial antifungal treatment before PCR and after PCR test.

	Before PCR test	After PCR test	Value of *p*
IAC	17/83 (20.5)	24^a^/83 (28.9)	<0.001
Antifungal initial treatment			<0.001
Preventive therapies	2/53 (3.8)	2/53 (3.8)	
Empirical therapies	42/53 (79.2)	33/53 (62.3)	
Preemptive therapies	3/53 (5.7)	1/53 (1.9)	
Target therapies	6/53 (11.3)	17/53 (32.1)	

Results are presented as *n* (%). ^a^Seven patients were assumed to be IAC because of positive *Candida* PCR in PF. PCR, polymerase chain reaction; IAC, intra-abdominal candidiasis.

There was no significant difference between the two groups except ICU LOS regarding the short-term outcomes of the two groups of patients (Table 8). The median ICU LOS in the IAC group was 16 days (5–23 days), which was significantly longer than that in the NIAC group (5 days [3–10.3 days]; *p* = 0.003).

**Table 8:** Outcomes analyses.

	Total (*N* = 83)	NIAC (*N* = 66)	IAC (*N* = 17)	Value of *p*
Overall mortality in ICU	10 (12.0)	7 (10.6)	3 (17.6)	0.42
Overall mortality in 28 days	11 (13.3)	9 (13.6)	2 (11.8)	1
Overall mortality in hospital	18 (21.7)	13 (19.7)	5 (29.4)	0.386
ICU LOS (d)	5 (3–13)	5 (3–10.3)	16 (5–23)	0.003
Hospital LOS (d)	27 (14–46)	25.5 (14–46)	38 (23–45.5)	0.153
Duration of mechanical ventilation (h)	66 (11–204)	65 (9–145.8)	114 (12.5–389)	0.196

Results are presented as *n* (%) and median (interquartile range). IAC, intra-abdominal candidiasis; LOS, length of stay.

## 4. Discussion

Our study found that the incidence of IAC accounted for 20.5% (17/83) of the high-risk population. Previous studies by Senn et al. (2009) showed that 30 to 40% of patients in a more specific high-risk population of IAC (recurrent gastrointestinal perforation/anastomotic leakage or acute necrotizing pancreatitis) developed IAC. Physicians should pay attention to patients at a high risk of IAC.

The multivariate logistic regression analysis demonstrated that surgical site involvement in the upper gastrointestinal tract and neutrophil percentage >90% at ICU admission were independent risk factors for IAC in the high-risk population. Hence, the risk of IAC is higher when the surgical site is the upper gastrointestinal tract. In addition, this study suggests that a neutrophil percentage >90% at ICU admission is a risk factor for IAC. Therefore, ICU staff should pay more attention to patients whose neutrophil percentage is >90% upon ICU admission.

In previous studies, PCR assay of blood samples for the diagnosis of IC had a sensitivity of 80–96.3% and specificity of 70–97.3% (Avni et al., 2011; Nguyen et al., 2012; Fortún et al., 2014). However, León et al. (2016) used PCR alone to detect *Candida* DNA in blood samples from ICU patients with severe abdominal disease, with a sensitivity of 84.0% and specificity of 32.9%. In the high-risk cohort of IAC in our study, only 11.8% (2/17) of IAC were diagnosed as IAC with candidemia, and IAC with candidemia accounted for 2.4% (2/83) of the high-risk population. This low proportion suggests that physicians should consider the feasibility of PCR in non-blood samples to avoid misdiagnosis IAC without candidemia. The significance of the blood PCR assay in patients without candidemia has been questioned. In the presence of IAC, PF reflects the local reality of the infectious site more directly. The process of *Candida* spp. entering the blood circulation is intermittent rather than continuous (Nguyen et al., 2012; Fortún et al., 2020), so it is more difficult to intercept *Candida* spp. in the blood than in PF. In addition, some studies have shown that fluid overload and hemodilution also reduce the sensitivity of PCR in blood samples (Nguyen et al., 2012; Fortún et al., 2020) but might have little effect on PF samples. In this study, the sensitivity and specificity of positive PCR for PF were 64.9 and 89.4%, respectively. Compared with the study by Fortún et al. (2020) in 2020, it was superior to the sensitivity of 25% in blood PCR, and the specificity was equivalent (89.4% vs. 90.9%). In a study by Fortún et al. (2020), a PCR assay was also performed on PF samples, which showed a false negative PCR result in 9 of the 17 cases with IAC; however, the specificity was high. The results of our trial also suggest that the specificity and negative predictive value of *Candida* PCR for PF had better performance in high-risk patients with IAC, providing a basis for the exclusion of IAC diagnosis. *Candida* PCR of PF in high-risk patients has important clinical significance; however, the lack of standardization and large sample size precludes its clinical use. This aspect deserves further investigation.

In this study, seven cases were positive for PCR in PF, but negative for PF direct microscopy examination, PF culture, and blood culture. 71.4% (5/7) patients received antifungal therapies, which were empirical and preemptive therapies. According to the current diagnostic gold standard (Bassetti et al., 2013), this group of patients was judged to have NIAC. Interpretation of the clinical significance of these patients remains controversial. The significance of positive PCR results in blood has been recognized (Soulountsi et al., 2021). PCR kits, such as the Light-Cycler SeptiFast and the IRIDA BAC BSI assay, have been used to detect microorganisms, including *Candida* spp., in blood to improve the early diagnosis of IC. However, the applicability of this method to PF samples remains uncertain. In Corrales et al. (2015) showed that the results of the PCR assay in PF were consistent with those of the automatic culture method and had potential clinical applications. The ESICM/ESCMID task force on the practical management of invasive candidiasis in critically ill patients (Martin-Loeches et al., 2019) recognized the excellent performance of PCR assay technology and suggested that NCBT, including PCR, should be combined with traditional culture technology as a diagnostic strategy. The present study assumed that positive PCR in samples would be included in the diagnostic criteria for IAC, which increased the incidence of IAC in high-risk groups from 20.5 to 28.9%, and that the proportion of initial target antifungal therapies would increase. Because the PCR assay takes less time, if this result is accepted, it would lead to earlier diagnosis and drive earlier targeted antifungal therapies, which may have clinical benefits. Therefore, large-sample multicenter prospective studies, including confirmed IAC cases, are needed to verify the diagnostic value of *Candida* PCR in PF for IAC and its driving effect on treatment. We have designed multiple detection primers of different *Candida* species genes, and hope to use them in the other study. We hope that we will be able to achieve rapid detection in the in-hospital laboratory in the future. This study also found that the early diagnostic efficacy of serum BDG alone was unsatisfactory, and the combination of PCR in PF and serum BDG improved the PPV. The combination of NCBT, such as BDG and PCR in PF for the early diagnosis of IAC requires further exploration in the future.

There were some limitations to this prospective trial. First, the PCR assay in this study used first-generation sequencing technology, which has its limitations. The *Candida* spectrum was narrow and did not cover all possible pathogenic *Candida* species. With the improvement of PCR technology, multiple real-time PCR technologies will be applied to PF samples. Second, to avoid the interference of colonization, the PF was collected within 24 h after placing the tube. The copy number of DNA might still be low at the time of sampling, which is not sufficient for detection. Third, this study was completed in a single center with a limited number of samples. Prospective multicenter studies are needed to confirm the efficiency of PCR assays in PF in the future.

In conclusion, this study suggests that PCR assay of *Candida* DNA in PF could be considered as an adjunct to existing routine diagnostic tools and optimize antifungal treatment of IAC in high-risk ICU patients. However, further multicenter research should be conducted to evaluate the performance of multiple real-time PCR in PF in IAC diagnosis, improve the sensitivity of PCR assays, and explore the effect of positive PCR on the antifungal treatment and prognosis of patients in high-risk populations.

## Data availability statement

The datasets presented in this study can be found in online repositories. The names of the repository/repositories and accession number(s) can be found at: https://www.ncbi.nlm.nih.gov/genbank/, OP418489 https://www.ncbi.nlm.nih.gov/genbank/, OP418490 https://www.ncbi.nlm.nih.gov/genbank/, OP418491 https://www.ncbi.nlm.nih.gov/genbank/, OP418492 https://www.ncbi.nlm.nih.gov/genbank/, OP418493 https://www.ncbi.nlm.nih.gov/genbank/, OP418494 https://www.ncbi.nlm.nih.gov/genbank/, OP418495 https://www.ncbi.nlm.nih.gov/genbank/, OP418496 https://www.ncbi.nlm.nih.gov/genbank/, OP418497 https://www.ncbi.nlm.nih.gov/genbank/, OP418498 https://www.ncbi.nlm.nih.gov/genbank/, OP418499 https://www.ncbi.nlm.nih.gov/genbank/, OP418500 https://www.ncbi.nlm.nih.gov/genbank/, OP418501 https://www.ncbi.nlm.nih.gov/genbank/, OP418502 https://www.ncbi.nlm.nih.gov/genbank/, OP418503 https://www.ncbi.nlm.nih.gov/genbank/, OP418504 https://www.ncbi.nlm.nih.gov/genbank/, OP418505 https://www.ncbi.nlm.nih.gov/genbank/, OP418506.

## Ethics statement

The studies involving human participants were reviewed and approved by The Clinical Research Ethics Committee of Peking University First Hospital. The patients/participants provided their written informed consent to participate in this study.

## Author contributions

MX contributed to the conceptualization, methodology, formal analysis, investigation, resource, data curation, writing–original draft, and funding acquisition. JS contributed to the conceptualization, methodology, investigation, and writing–original draft. ZW contributed to the methodology, investigation, and resource. TY contributed to the conceptualization, methodology, investigation, formal analysis, and project administration. SZ contributed to the methodology, formal analysis, resource, data curation, writing–review and editing. SL contributed to the conceptualization, methodology, formal analysis, writing–review and editing, and supervision. JY contributed to the conceptualization, methodology, writing–review and editing, and supervision. All authors contributed to the article and approved the submitted version.

## Funding

This work was supported by the Seed Fund Project of Peking University First Hospital [2018SF083], National High Level Hospital Clinical Research Funding (Interdisciplinary Clinical Research Project of Peking University First Hospital [2022CR43]) and Nation Key Research and Development Project [2020YFC2005401].

## Conflict of interest

The authors declare that the research was conducted in the absence of any commercial or financial relationships that could be construed as a potential conflict of interest.

## Publisher’s note

All claims expressed in this article are solely those of the authors and do not necessarily represent those of their affiliated organizations, or those of the publisher, the editors and the reviewers. Any product that may be evaluated in this article, or claim that may be made by its manufacturer, is not guaranteed or endorsed by the publisher.
