# Supplementary material for: Detection of Candida DNA in peritoneal fluids by PCR assay optimizing the diagnosis and treatment for intra-abdominal candidiasis in high-risk ICU patients: A prospective cohort study
Source: Front Microbiol. 2023 Jan 5;13:1070688. doi: 10.3389/fmicb.2022.1070688 (PMC9849671; doi:10.3389/fmicb.2022.1070688)
Supplement: Supplementary file 1 [file Table_1.docx]

Supplementary Material

# Supplementary Tables

## Supplementary Table 1

**SUPPLEMENTARY TABLE 1. High-risk factors for IAC in upper and non-upper gastrointestinal tract group**

1. **Upper gastrointestinal tract group (N=23)**

| **High-risk factors for IAC** | **Total (N=23)** | **NIAC (N=16)** | **IAC (N=7)** | **Value of *P*** |
| --- | --- | --- | --- | --- |
| Recurrent gastrointestinal perforations, perforations untreated for more than 24 hours, or both | 12 (52.2) | 7 (43.8) | 5 (71.4) | 0.371 |
| Recurrent abdominal surgery within 30 days | 7 (30.4) | 6 (37.5) | 1 (14.3) | 0.366 |
| Postoperative suspected or confirmed gastrointestinal anastomosis leakage | 5 (21.5) | 4 (25.0) | 1 (14.3) | 1.000 |
| Septic shock | 10 (43.5) | 7 (43.8) | 3 (42.9) | 1.000 |

1. **Non-upper gastrointestinal tract group (N = 60)**

| **High-risk factors for IAC** | **Total (N=60)** | **NIAC (N=50)** | **IAC (N=10)** | **Value of *P*** |
| --- | --- | --- | --- | --- |
| Recurrent gastrointestinal perforations, perforations untreated for more than 24 hours, or both | 27 (45.0) | 21 (42.0) | 6(60.0) | 0.322 |
| Recurrent abdominal surgery within 30 days | 13 (21.7) | 11 (22.0) | 2 (20.0) | 1.000 |
| Postoperative suspected or confirmed gastrointestinal anastomosis leakage | 16 (26.7) | 12 (24.0) | 4 (40.0) | 0.433 |
| Septic shock | 36 (60.0) | 31 (62.0) | 5 (50.0) | 0.501 |

Results are presented as n (%). IAC, intra-abdominal candidiasis.
